# Analysis of the Bacterial Communities in Two Liquors of Soy Sauce Aroma as Revealed by High-Throughput Sequencing of the 16S rRNA V4 Hypervariable Region

**DOI:** 10.1155/2017/6271358

**Published:** 2017-02-28

**Authors:** Jing Tang, Xiaoxin Tang, Ming Tang, Ximin Zhang, Xiaorong Xu, Yin Yi

**Affiliations:** ^1^The Key Laboratory of Plant Physiology and Development in Guizhou Province, Guizhou Normal University, Guiyang, Guizhou Province 550001, China; ^2^College of Bioscience, Guizhou Normal University, Guiyang, Guizhou Province 550001, China

## Abstract

Chinese liquor is one of the world's oldest distilled alcoholic beverages and an important commercial fermented product in China. The Chinese liquor fermentation process has three stages: making* Daqu* (the starter), stacking fermentation on the ground, and liquor fermentation in pits. We investigated the bacterial diversity of Maotai and Guotai* Daqu* and liquor fermentation using high-throughput sequencing of the V4 hypervariable region of the 16S rRNA gene. A total of 70,297 sequences were obtained from the* Daqu* samples and clustered into 17 phyla. The composition of the bacterial communities in the* Daqu* from these two soy sauce aroma-style Chinese liquors was the same, although some bacterial species changed in abundance. Between the* Daqu* and liquor fermentation samples, 12 bacterial phyla increased. The abundance of* Lactobacillus* and* Pseudomonas* increased in the liquor fermentation. This study has used high-throughput sequencing to provide new insights into the bacterial composition of the Chinese liquor Daqu and fermentation. Similarities in the distribution of bacteria in the soy sauce aroma-style Chinese liquors* Daqu* suggest that the abundance of bacteria might be generally concerned to other liquor.

## 1. Introduction

Fermentation is a well-known ancient technique that uses microorganism to process and preserve food. Chinese liquor is one of the six well-known distillates in the world. It has a long history of production and is produced through unique a fermentation process. It is typically produced from cereals, such as sorghum and rice, via the solid-state fermentation of grain. Chinese liquor has five main styles: strong aroma, light aroma, soy sauce aroma, sweet honey, and miscellaneous.* Maotai* and* Guotai* are Chinese liquors famous for their soy sauce aroma.* Maotai-flavor* liquor is as symbolic a drink in China as whisky is in Scotland and brandy in France [1–3].

Microorganisms usually do the main work of degrading biopolymers, producing alcohol, and forming aromatic compounds. The microbial community of Chinese liquor has been analyzed in previous studies using culture-dependent and culture-independent methods. Culture-dependent studies of the microbial community have used methods such as isolation and enumeration on selective media [4–6]. Culture-independent studies have used methods such as polymerase chain reaction-denaturing gradient gel electrophoresis (PCR-DGGE) [7–9], amplified fragment length polymorphism [10], and 16S RNA or 26S RNA clone libraries [11].

**Figure 1:**
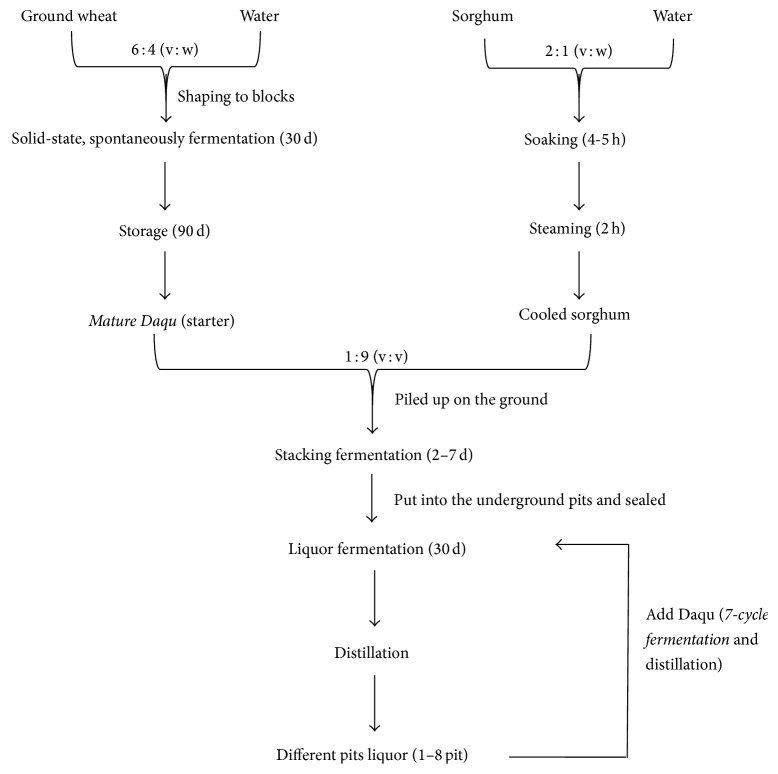
Flow sheet for making soy sauce aroma-style flavor liquor.

Chinese liquors are typically produced via solid-state fermentation using a natural fermentation starter termed* Daqu*. The* Daqu* starter has long been believed to play a key role in the fermentation of Chinese liquor [12].* Maotai* and* Guotai* liquors share the same unique and complicated spontaneous fermentation process, which includes making* Daqu *(the starter), stacking fermentation, and liquor fermentation (Figure 1) [13]. In the* Daqu*-making stage, the maximum temperature of* Daqu* reaches approximately 65°C, which is typical of high-temperature* Daqu*. Several studies have shown the diversity of the microbial community in* Daqu* or fermentation, especially the yeast and fungi [2, 3, 13, 14]. Therefore, it was of interest to analyze the microbial community in* Daqu* and find the bacteria important to* Daqu* properties. Little is known regarding the bacterial community composition in* Maotai Daqu*.

In previous research, culture-independent cloning methods were used for the analysis of microbial communities in* Daqu* or Chinese liquor fermentation. Here, we applied high-throughput sequencing of the V4 hypervariable region of the 16S rRNA gene to examine in-depth microbial communities from soy sauce aroma-style Chinese liquor to gain insight into the specific fermentative microorganisms. The main objective of this study was to (i) analyze the composition of the microbial communities in the* Daqu* of soy sauce aroma-style Chinese liquor; (ii) compare them with two different* Daqu* of soy sauce aroma-style Chinese liquor; and (iii) compare the composition of the microbial communities in the* Daqu* with those in the liquor fermentation.

## 2. Materials and Methods

### 2.1. Samples of* Daqu* and Fermented Grains

Sampling was performed in two different liquor production factories (*Maotai* and* Guotai*) in Guizhou Province, China. Three pits of* Daqu* samples from* Maotai* and* Guotai* were taken in the same years from 2011 to 2013. Samples of fermented grains were obtained from a randomly selected fermentation batch at the same time points (the fourth liquor fermentation) in 2013. The sampling was randomly selected from the mixture of the upper, middle, and bottom stacked layers. All samples were transferred to sterile bags, sealed, and stored at −80°C.

### 2.2. DNA Extraction and Quantitation

10 g samples were suspended in 50 mL of sterile PBS buffer (0.1 mol/L, pH 7.2–7.4) and vortexed for 15 min at ambient temperature. The suspension was then centrifuged (500 ×g, 4°C) for 5 min, and the pellet was washed three times in PBS buffer. The supernatants were also collected and centrifuged (10,000 ×g, 4°C) for 10 min; the resulting pellets were washed three times in PBS buffer. The resulting pellets were resuspended in PBS buffer and stored at −20°C until DNA extraction. DNA was extracted according to the method of Wang et al. [2, 3].

### 2.3. MiSeq Sequencing of 16S rRNA Gene Amplicons

The communities of bacteria were analyzed using Illumina MiSeq sequencing of the 16S rRNA gene V4 region amplicons, which can yield accurate taxonomic information and shows few biases for various bacterial taxa [15]. The V4 region of the 16S rRNA gene was amplified with the primer set 515f (5′-GTGCCAGCMGCCGCGGTAA-3′)/806r (5′-GGACTACHVGGGTWTCTAAT-3′), and all PCR amplifications were conducted in triplicate for each sample. This short targeted gene region can provide sufficient resolution for the accurate taxonomic classification of microbial sequences [16]. The initial 10 cycles of PCR amplification were performed. The products were then purified with Agencourt® Ampure® XP (Beckman Coulter, Inc., CA, USA) and used as a template for the second PCR amplification of 20 cycles using the same primer set; however, the reverse primer contained the appropriate adapters and different barcodes to distinguish samples. PCR products were visualized using 1% agarose gels stained with ethidium bromide, and negative controls were always performed to confirm the absence of contamination. True positive amplicons were quantified using a PicoGreen dsDNA Assay kit (Invitrogen, CA, USA), combined equally, and then gel purified. The DNA library was sequenced using the Illumina MiSeq platform according to the manufacturer's instructions [17]. Sequences were analyzed with the QIIME [18] software package and UPARSE pipeline [19]. Quality filtering and processing of MiSeq reads were conducted by QIIME. Default settings for Illumina processing in QIIME were used (*r* = 3  *p* = 0.75 total read length; *q* = 3; *n* = 0) (*p*: minimum number of consecutive high-quality base calls to retain read; *r*: maximum number of consecutive low-quality base calls allowed before truncating a read; *n*: maximum number of ambiguous (*N*) characters allowed in a sequence; *q*: last quality score considered low quality). We ultimately obtained 10,083–17,973 high-quality sequences from the* Daqu* samples and 12,418–15,302 sequences from the liquor fermentation samples. Then we use UPARSE pipeline to picking operational taxonomic units (OTUs) through making OTU table. Sequences were assigned to OTUs at 97% similarity. We pick representative sequences for each OTU and use the RDP classifier [20] to assign taxonomic data to each representative sequence.

### 2.4. Statistical Analysis

The datasets generated using 16S rRNA gene sequencing (OTU composition) were further analyzed with the following statistical methods: (i) *α*-diversity comparison (Chao value and Shannon index) and *β*-diversity comparison (unweighted UniFrac distances and weighted UniFrac distances); (ii) hierarchical clustering based on the relative abundance of bacteria (the specificity measure (SPM)) and Euclidean distance and complete linkage being used in this clustering analysis; and (iii) significance tests based on unpaired Student's* t*-tests and Wilcoxon rank-sum test to identify differences between any two compared objects. All statistical analyses described above were performed using the R package vegan/gplot. To quantitatively estimate the relative abundance of a bacterial genus in a sample, the specificity measure (SPM) [21] was introduced as follows and was used in a Heatmap. Each abundance of a bacterial genus was first transformed into vector *X*:

(1) $$\begin{array}{c} X=\left(\;x_{1},x_{2},\ldots ,x_{i},\ldots ,x_{n-1},x_{n}\;\right), \end{array}$$

where *n* is the number of samples in a profile. At the same time, vector *X*_*i*_ was created to represent the abundance of a bacterial genus in sample *i*:

(2) $$\begin{array}{c} X_{i}=\left(\;0,0,\ldots ,x_{i},\ldots ,0,0\;\right). \end{array}$$

The SPM of a bacterial genus in a sample was then determined by calculating the cosine value of intersection angle *θ* between vectors *X*_*i*_ and *X* in high-dimension feature space:

(3) $$\begin{array}{c} {SPM}_{i}=\cos\theta =\frac{X_{i}\cdot X}{\left|X_{i}\right|\cdot \left|X\right|}, \end{array}$$

where |*X*_*i*_| and |*X*| are the length of vectors *X*_*i*_ and *X*, respectively. The value of SPM ranges from 0 to 1.0. A SPM value close to 1.0 indicates the major contribution of bacterial abundance in a designated sample (e.g., vector *X*_*i*_) against that in all samples (vector *X*).

## 3. Results

### 3.1. Composition of Bacterial Communities in Different* Daqu* Determined Using High-Throughput Sequencing

**Figure 2:**
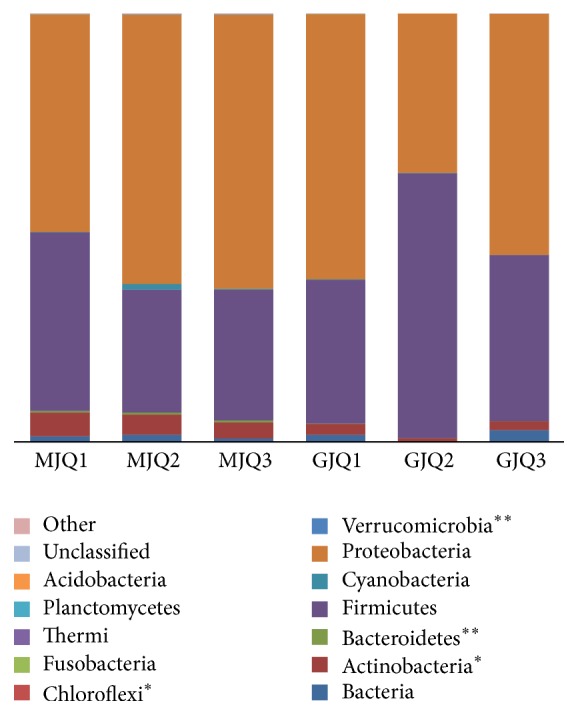
The percentages of OTUs assigned to major bacterial phyla (≥0.01%). Three pits of Maotai-liquor Daqu samples in 2011 to 2013 years are abbreviated as MJQ1, MJQ2, and MJQ3, respectively, and the three Guotai-liquor Daqu samples are GJQ1, GJQ2, and GJQ3 (*t*-test, ^*∗*^*p* < 0.05, ^*∗∗*^*p* < 0.01). An asterisk indicates a significant difference with major bacterial phyla between the Maotai-liquor Daqu and Guotai-liquor Daqu.

**Figure 3:**
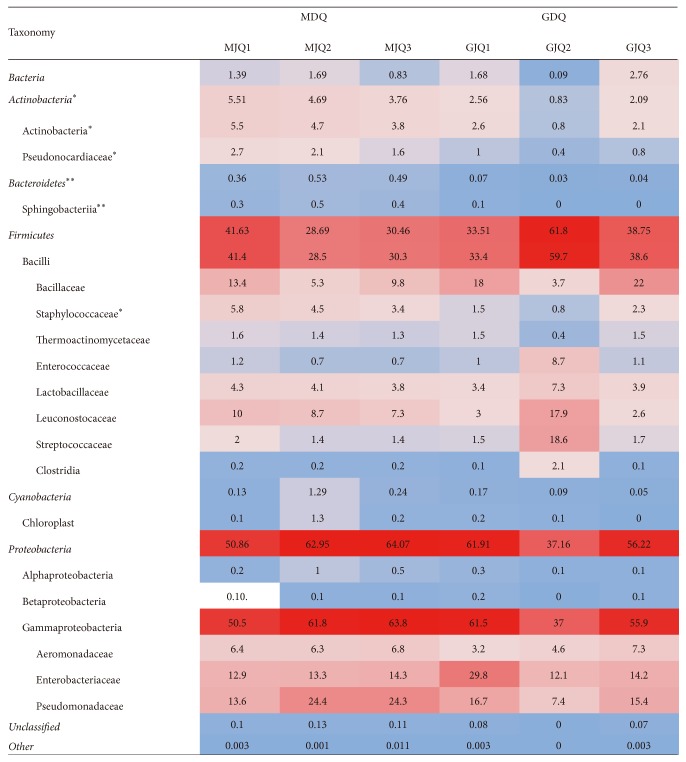
Abundance of the dominant taxonomy (relative abundance of phylum > 0.1% and of family > 1.0%) determined by 16S rRNA gene sequencing. Numbers in cells are percentages of relative abundance, highest abundances are red, middle values are in white, and lowest values are in blue. An asterisk indicates a significant different between the Guotai-liquor Daqu and the Maotai-liquor Daqu. (*t*-test, ^*∗*^*p* < 0.05;^*∗∗*^*p* < 0.01).

A total of 70,297 high-quality sequences (approximately 260 bp) were obtained from the 6* Daqu* samples, with an average of 11,716 sequences per sample. There were no significant differences between the* Maotai Daqu* high-quality sequences with the* Guotai Daqu *(*t*-test, *p* = 0.530). The OTUs detected in the* Guotai Daqu* largely overlapped with the* Maotai Daqu*; the number of OTUs in each of the 6 samples (average 5,418) was similar (*t*-test, *p* = 0.691). These OTUs clustered into 17 phyla, 55.5% of which, on average, were classified as Proteobacteria, followed by Firmicutes 39.1% (Figure 2). In terms of relative abundance, Proteobacteria (mainly Gammaproteobacteria) were the most abundant bacterial phylum in the Chinese liquor* Daqu* (accounting for 37.16–64.07% of the different* Daqu* samples), and Firmicutes (mainly Bacilli) were also abundant (accounting for 28.69–61.80%; Figure 3). There were no significant differences (*t*-test, *p* > 0.05) in the most bacterial relative abundances with two* Daqu* samples. However, Actinobacteria and Bacteroidetes were significantly different (*t*-test, *p* < 0.05). Of all the bacterial families, Bacillaceae, Enterobacteriaceae, and Pseudomonadaceae were the most abundant.

The Chao value and Shannon index, which reflect the *α*-diversity of bacterial communities, showed no significant differences between the* Guotai Daqu* and the* Maotai Daqu* (*t*-test, *p* = 0.140 and *p* = 0.117, and Wilcoxon rank-sum test, *p* = 0.210 and *p* = 0.213, resp.). The unweighted UniFrac distances and weighted UniFrac distances which reflect the *β*-diversity of bacterial communities showed no significant differences between the* Guotai Daqu* and the* Maotai Daqu* (Wilcoxon rank-sum test, *p* = 0.128 and *p* = 0.462) (Table 1).

**Figure 4:**
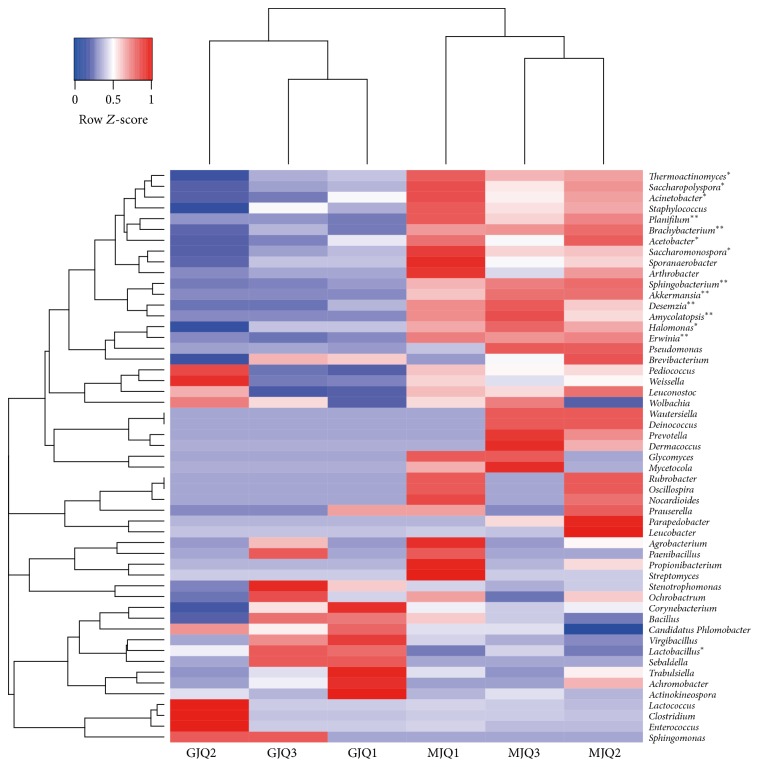
Heatmap showing the differences among the six investigated communities based on the abundance of the domain genura. (relative abundance > 0.01%) An asterisk indicates a significant different between Goutai and Maotai Daqu. (*t*-test, ^*∗*^*p* < 0.05, ^*∗∗*^*p* < 0.01).

However, the 14 genera whose abundances were greater than 0.01% of the total bacteria in the* Guotai *and the* Maotai* were significantly different (*t*-test, *p* < 0.05; Figure 4). In terms of relative abundance, 13 bacterial genera were significantly higher in the* Maotai Daqu*, and only* Lactobacillus* was significantly higher in the* Guotai Daqu*. Although there were no significant differences in the number of detected bacterial taxa, the samples clustered into two groups based on the relative abundances of the major genera, indicating clear differences between* Maotai Daqu* and* Guotai Daqu *(Figure 4). Including* Thermoactinomyces*,* Saccharopolyspora*,* Acinetobacter,* and* Pseudomonas*, 17 bacterial genera in the* Maotai Daqu* were higher, and 6 genera bacterial (from* Corynebacterium* to* Sebaldella*) in the* Guotai Daqu* were higher.

### 3.2. Composition of Bacterial Communities in Liquor Fermentation Determined Using High-Throughput Sequencing

**Figure 5:**
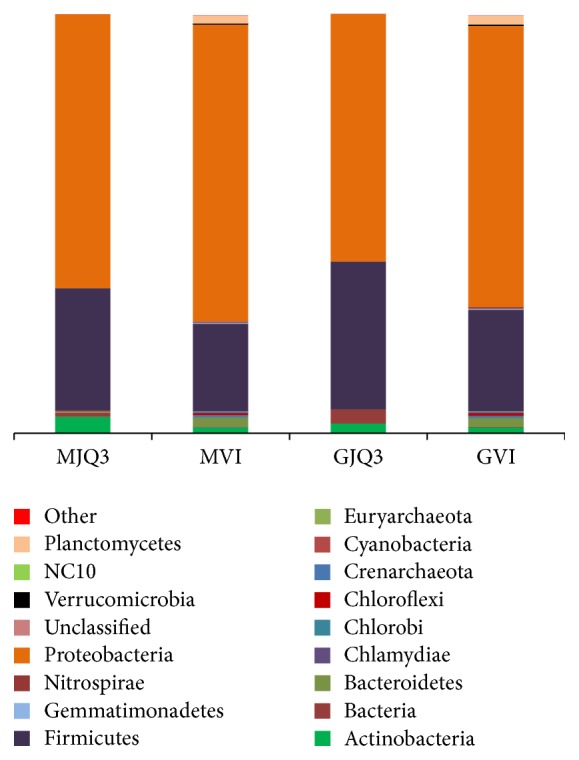
The percentages of OTUs assigned to major bacterial phyla (≥0.01%). The Maotai-liquor fermentation sample are abbreviated as MVI, and the Guotai-liquor fermentation as GVI. The Daqu sample in 2013 are abbreviated as MJQ3 and GJQ3.

A total of 27,720 high-quality sequences were obtained from the 2 liquor fermentation samples, corresponding to 10,200 OTUs. These OTUs clustered into 27 phyla, the most abundant of which was Proteobacteria (67.04% and 70.76%), followed by Firmicutes (20.74% and 24.11%, Figure 5). The OTUs clustered into 17 phyla in the* Daqu* sample and then in the liquor fermentation sample plus an additional 10 phyla. Twelve bacterial phyla were more abundant in the liquor fermentation process, including Acidobacteria, Bacteroidetes, Chlorobi, Chloroflexi, Proteobacteria, and Planctomycetes. However, Actinobacteria and Firmicutes were less abundant in the liquor fermentation process relative to the* Daqu* samples.

**Figure 6:**
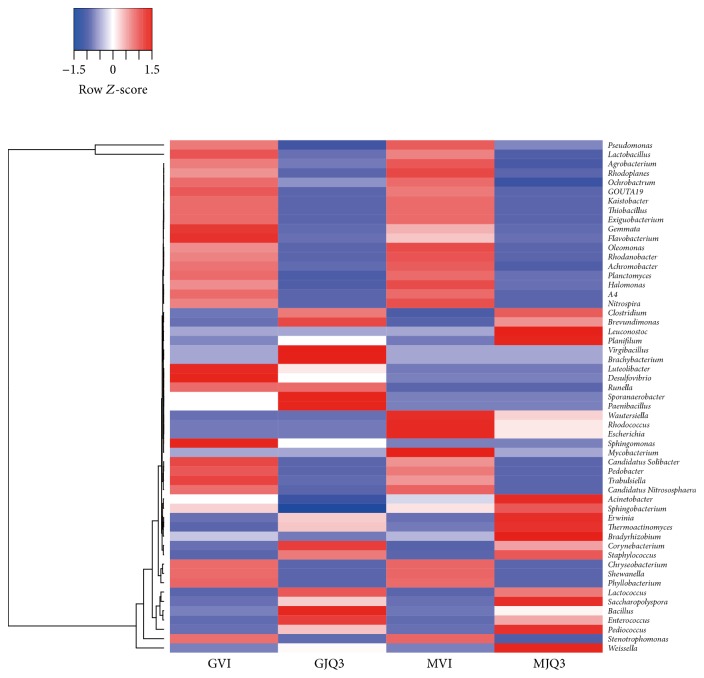
Heatmap showing the difference among the liquor fermentation based on the abundance of the domain genera. (relative abundance > 0.01%).

These OTUs clustered into 314 genera, and the genera whose abundances were greater than 0.01% of the total bacteria were different between the Daqu and liquor fermentation samples (Figure 6).* Lactobacillus *abundance increased from an average of 1.35% to 19.78% in the liquor fermentation and* Pseudomonas *from 5.07% to 33.52%. In the liquor fermentation process, 18 bacterial genera increased, including* Pseudomonas*,* Lactobacillus*,* Agrobacterium*,* Rhodoplanes*,* Ochrobactrum*, and* Nitrospira*, and 5 bacterial genera decreased,* Lactococcus*,* Enterococcus*,* Saccharopolyspora*,* Bacillus*, and* Pediococcus*.

## 4. Discussion

Prior studies on the Chinese liquor fermentation process have focused on a limited number of isolated samples or microbial diversity examined using 16S rRNA gene library analysis and PCR-DGGE. Previously, the higher bacterial diversity as measured by the Shannon index (*H*′ = 1.19) was found in a high-temperature Daqu (9-H-S-W) [11]. In the current study, the average Shannon index of the* Daqu* was *H'* = 6.58. Using high-throughput sequencing of the V4 hypervariable region of the 16S rRNA gene to examine microbial communities in-depth, the abundance of the bacterial community in* Daqu* was higher than previously reported [2, 3, 6, 9]. Some of the bacterial genera observed in our study were not previously reported.

Knowledge of the microbiota of Chinese liquor fermentation is still far from complete, especially the microbiota of* Daqu*. Therefore, this study was initiated to understand the composition of the microbial community in two representative soy sauce aroma-style Chinese liquors. In the soy sauce aroma-style Chinese liquor,* Daqu* is made from ground wheat and is produced in Guizhou Province in China, applying high-temperature fermentation conditions for* Daqu *production. It is expected that the relative abundance of several of the microorganisms identified in the* Daqu* correlate with different Chinese liquors. Several studies indicated that the bacterial community in Daqu is affected by certain factors, including raw materials, environmental conditions (e.g., soil and air), moisture content, oxygen condition and “mother* Daqu*” (Daqu that was produced 1 year ago).* Maotai*-flavor and* Guotai*-flavor liquor have the same unique, complicated spontaneous fermentation process and the same environmental conditions. Their* Daqu* may thus have the same microbial communities. The Chao value and Shannon index, which reflect the *α*-diversity of bacterial communities, of the* Daqu* samples were not significantly different between the* Guotai Daqu* and the* Maotai Daqu*. Furthermore, the same bacterial species were present in the* Daqu* of both liquors. However, the abundance of 13 bacterial genera in the* Maotai Daqu* was significantly higher than in the* Guotai Daqu*:* Thermoactinomyces*,* Erwinia, Saccharopolyspora*,* Acinetobacter*,* Planifilun*,* Brachybacterium*,* Acetobacter*,* Akkermansia*,* Saccharomonospora*,* Sphingobacterium*,* Desemzia*,* Amycolatopsis*, and* Halomonas*. Only the abundance of* Lactobacillus* was significantly higher in the* Guotai Daqu* than in the* Maotai Daqu *(Figure 4). Based on our analysis of different* Daqu* samples from soy sauce aroma-style Chinese liquor, we concluded that the composition of the bacterial communities in the different Chinese liquor were the same. Nevertheless, some bacterial species have significantly different abundances between the different* Daqu* samples. Some types of* Daqu *contained highly similar bacteria species, whereas some bacterial species abundances were significantly different. The relative abundance of bacteria may be important for the Daqu.

Several studies mentioned the importance of lactic acid bacteria (LAB) during the production of* Daqu*, including* Enterococcus*,* Lactobacillus*,* Leuconostoc*,* Pediococcus*,* Streptococcus*, and* Weissella*. In this study, various genera of lactic acid bacteria (LAB) were identified in* Maotai* and* Guotai Daqu*. In the current study, LAB were only found at high abundance during the beginning of the* Fenjiu-Daqu* production process [22], and LAB except* Weissella *were found in low abundance [11]. We observed a high abundance of* Weissella *and* Pediococcus*, and the abundance of LAB except* Lactobacillus *was not significantly different. Some species of* Lactobacillus* inhibit the growth of* Bacillus* [23]. However, in this study, there were no significant differences in* Bacillus* between the two* Daqu *samples.* Bacillus* is a well-known producer of proteases and amylases [24] and produces more than 70 metabolites, most of which are flavor compounds and flavor precursors (Yan et al. 2013). The production of these molecules is important for the aroma of fermented products. This explained that the abundance of* Bacillus* in* Maotai* and* Guotai Daqu *was not significantly different, although* Lactobacillus *was. In the liquor fermentation process, the abundance of LAB excluding* Lactobacillus *decreased, and* Lactobacillus *abundance increased from the average 1.35% to 19.78%. With the dramatic increase of* Lactobacillus*, the abundance of* Bacillus *decreased. At the initial stage of stacking fermentation,* Lactobacillus* quickly propagates and becomes the main bacteria [2, 3].

In this study, the abundance of the phyla Actinobacteria, Bacteria, and Firmicutes decreased between both types of* Daqu* and the fermentation processes, while Proteobacteria and others increased. The genus* Lactobacillus* of the family Lactobacillaceae and the* Pseudomonas *of the family Pseudomonadaceae increased. When the fermentation began, several chemicals were enriched, making the habitat suitable for some bacteria, such as* Lactobacillus* and* Pseudomonas*. During the Fen liquor fermentation process, the bacterial community diversity decreases, and only the family Lactobacillaceae increases [25]. The family Lactobacillaceae is a contributor to the fermentation reaction.

Different microbial communities in Daqu facilitate the selection of starters for creation of unique flavors. Further research is required to gain deeper insight into the microbial communities of the different types of Daqu and the function of unknown microorganisms in these communities. This work may increase liquor producers' understanding of the bacterial community in* Daqu*, and the relative abundance of bacteria in Daqu may be another important topic for liquor production.

## Figures and Tables

**(a):** 

a-diversity	MJQ1	MJQ2	MJQ3	GJQ1	GJQ2	GJQ3	*p*(*t*−)	*p*(*w*−)
Chao value	3655.8	3872.88	3427.75	3038.48	3067.18	3608.63	0.140	0.210
Shannon index	6.782	6.876	6.863	6.199	5.944	6.818	0.117	0.213

**(b):** 

	MJQ1	MJQ2	MJQ3	GJQ1	GJQ2	GJQ3
MJQ1	0	0.119^*∗*^	0.187^*∗*^	0.200^*∗*^	0.190^*∗*^	0.183^*∗*^
MJQ2	0.315^*∗∗*^	0	0.201^*∗*^	0.177^*∗*^	0.178^*∗*^	0.189^*∗*^
MJQ3	0.397^*∗∗*^	0.418^*∗∗*^	0	0.171^*∗*^	0.141^*∗*^	0.181^*∗*^
GJQ1	0.441^*∗∗*^	0.434^*∗∗*^	0.404^*∗∗*^	0	0.081^*∗*^	0.172^*∗*^
GJQ2	0.457^*∗∗*^	0.465^*∗∗*^	0.411^*∗∗*^	0.302^*∗∗*^	0	0.171^*∗*^
GJQ3	0.465^*∗∗*^	0.469^*∗∗*^	0.396^*∗∗*^	0.327^*∗∗*^	0.308^*∗∗*^	0
